# A Proanthocyanidins-Rich Cili (*Rosa roxburghii*) Fruit Extract Protects CCl_4_-Induced Mouse Hepatic Fibrosis via Modulation of Ferroptosis and Gut Microbiota

**DOI:** 10.3390/nu17213463

**Published:** 2025-11-03

**Authors:** Yang Liu, Jingzhong Zheng, Xin Zheng, Dan Zhou, Hang Ma, Xue Zhou, Fahuan Ge

**Affiliations:** 1 School of Pharmaceutical Sciences, Sun Yat-sen University, Guangzhou 510006, China; 2 Bioactive Botanical Research Laboratory, Department of Biomedical and Pharmaceutical Sciences, College of Pharmacy, The University of Rhode Island, Kingston, RI 02881, USA

**Keywords:** Cili (*Rosa roxburghii*), proanthocyanidins, liver fibrosis, ferroptosis, TGF-β1/Smad3, gut microbiota

## Abstract

**Background:** Cili (*Rosa roxburghii* Tratt) is a unique fruit native to China’s Yunnan–Guizhou Plateau, rich in vitamin C, polyphenols, and triterpene, with broad health-promoting effects. Although cili’s hepatoprotective properties are reported, the bioactive components and underlying mechanisms remain poorly defined. **Methods:** We enriched proanthocyanidins from cili using column chromatography, identified their components via UPLC-Q-TOF-MS/MS, and validated their anti-liver fibrosis effects through in vitro and in vivo experiments. **Results:** Herein, we developed a novel proanthocyanidin-rich cili fruit extract (PACs-CFE) containing 84.2% total proanthocyanidins, comprising catechins, epicatechins, and diverse B-type dimers, trimers, tetramers, and gallate esters, as characterized by UPLC-Q-TOF-MS/MS. PACs-CFE inhibited LX-2 activation, suppressed collagen III and α-SMA expression, and induced ferroptosis via mitochondrial injury, reactive oxygen species accumulation, and GPX4/ferritin downregulation. In vivo, PACs-CFE ameliorated liver fibrosis, restored hepatic architecture, and improved serum alanine aminotransferase, aspartate aminotransferase, and bilirubin profiles. Moreover, PACs-CFE modulated the TGF-β1/Smad3 signaling pathway and beneficially reshaped the gut microbiota, enriching anti-inflammatory and hepatoprotective genera while reducing pathogenic taxa. **Conclusions:** Our findings show that PACs-CFE exerts multi-targeted anti-fibrotic effects through hepatic stellate cell inactivation, ferroptosis induction, TGF-β1/Smad3 suppression, and gut–liver axis modulation. This study provides useful insight into the hepatoprotective potential of cili fruit and supports its development as standardized functional ingredients for liver health.

## 1. Introduction

Cili (*Rosa roxburghii* Tratt), known as chestnut rose or burr rose, is a unique medicinal and edible plant native to China’s Guizhou Province, where it thrives in the ecologically diverse and often harsh environment of the Yunnan-Guizhou Plateau [1]. This high-altitude region, characterized by strong ultraviolet exposure and poor soil conditions, contributes to the plant’s adaptive resilience and rich phytochemical profile. Traditionally consumed as fruit and juice, cili has attracted growing scientific interest due to its abundant bioactive compounds, notably vitamin C, polyphenols, and carotenoids, which collectively contribute to its strong antioxidant capacity [2]. Beyond its antioxidant capacity, these bioactive compounds are associated with a broad range of health-promoting effects, including anti-inflammatory, anti-tumor, cardioprotective, hypoglycemic, and hepatoprotective activities [3,4,5,6]. Among these biological effects, the hepatoprotective activity of cili fruit has garnered increasing attention due to the rising prevalence of chronic liver diseases. Several preclinical studies suggest that cili fruit extracts may ameliorate liver injury by reducing oxidative stress and modulating inflammatory pathways [4,7,8]. However, the specific bioactive constituents responsible for these effects remain poorly defined.

Liver fibrosis is a progressive pathological condition characterized by excessive accumulation of extracellular matrix (ECM) in response to chronic liver injury. Drug-induced liver injury is the most common cause of acute liver failure in the Western world, and a significant proportion of commonly used medications can cause liver damage such as paracetamol, NSAIDs and tramadol [9]. If left untreated, fibrosis can disrupt normal liver architecture, induce portal hypertension, and ultimately progress to hepatocellular carcinoma (HCC), a leading cause of cancer-related mortality worldwide [10,11]. A key driver of fibrogenesis is the activation of hepatic stellate cells (HSCs), which transdifferentiate into myofibroblast-like cells that secrete ECM components and promote the formation of fibrotic scar [12]. Natural products, particularly plant-derived antioxidants such as phenolic compounds, have shown considerable promise in modulating key pathways involved in liver fibrosis [13,14,15]. These compounds may exert hepatoprotective effects by attenuating oxidative stress, inhibiting hepatic stellate cell activation, and regulating pro-fibrotic signaling cascades [13,14,15]. These compounds, based on their inherent low toxicity and hepatoprotective effects, can mitigate the hepatotoxic side effects of drugs. Therefore, developing it into a hepatoprotective agent holds significant importance.

Our research group previously reported that a cili fruit extract (CFE) prepared via low-temperature extraction protected liver cells against oxidative stress and fibrosis [7]. Although CFE contains a high total polyphenol content, it remains unclear whether specific phenolic subclasses, such as proanthocyanidins (PACs), are primarily responsible for its hepatoprotective effects. Moreover, the underlying mechanisms by which CFE exerts its liver protective activity have not been fully elucidated. Herein, we developed a novel proanthocyanidin-rich cili fruit extract (PACs-CFE) using macroporous resin column chromatography. The phytochemical composition of PACs-CFE was characterized by ultra-performance liquid chromatography coupled with quadrupole time-of-flight mass spectrometry (UPLC-Q-TOF-MS/MS). Its hepatoprotective effects were evaluated through a combination of in vitro assays using human hepatic stellate cells and in vivo models of thioacetamide-induced liver injury in zebrafish and carbon tetrachloride-induced fibrosis in mice. Furthermore, we explored the underlying mechanisms of PACs-CFE’s action by examining its impact on ferroptosis, TGF-β1/Smad3 signaling, and gut microbiome.

## 2. Materials and Methods

### 2.1. Experimental Materials and Reagents

Fresh cili fruits (Guinong-5) were collected from Bijie City, Guizhou Province, China, in 2022. A voucher specimen (CL-GN3-22) is deposited in the School of Pharmaceutical Sciences at Sun Yat-sen University (Guangzhou, Guangdong, China). Chromatographic-grade acetonitrile, formic acid, and phosphoric acid were purchased from Sucheng Yue Trading (Guangzhou, Guangdong, China). Proanthocyanidin standards were obtained from McLean Biochemistry Technology (Shanghai, China). (+)-Catechin was purchased from Yuanye Biotechnology (Shanghai, China), and epicatechin was from Vickiqi Biotech (Chengdu, Sichuan, China). Procyanidin B1 was purchased from Aladdin Scientific (Shanghai, China). Carbon tetrachloride (CCl_4_) and thioacetamide (TAA) were obtained from McLean Biochemistry Technology, Shanghai, China and Aladdin Scientific, Shanghai, China, respectively. Colchicine was supplied by Aladdin Scientific, Shanghai, China. Reticulin fiber staining kit (Modified Gomori’s), Masson trichrome staining kit, and CCK-8 (Goonie, 100–106) were purchased from Solarbio Science & Technology (Beijing, China). Human recombinant TGF-β1 (HEK293 derived) protein was obtained from PeproTech (Waltham, MA, USA).

### 2.2. Preparation of PACs-Enriched Cili Fruit Extract

Fresh cili fruits (10 kg) were pressed for juice and the juice extract was centrifuged and filtered. The filtrate was freeze-dried using a freeze-dryer (Xiangyi H1850-R-Cence; Changsha, China), and the powder was subjected to purification using an LX-38 macroporous resin column (Henghuibio®, Beijing, China). The column was first eluted with distilled water (3× column-volume) and then 30% aqueous ethanol (3× column-volume). The ethanol elute was then lyophilized to afford a PACs-rich cili fruit extract (PACs-CFE).

### 2.3. Measurement of PACs Content

Proanthocyanidin content was determined using a modified Porter method [16]. Briefly, proanthocyanidin standard solutions and PACs-CFE samples were prepared in methanol. Each solution (1 mL) was mixed with 6.0 mL of n-butanol–HCl (95:5, *v*/*v*) and 0.2 mL of ammonium ferric sulfate in a test tube. The mixture was heated in boiling water for 40 min, cooled rapidly, and the absorbance of each sample was measured at a wavelength of 550 nm using a spectrophotometer (Shimadzu UV-2600, Shimadzu, Kyoto, Japan), with methanol solution as the blank. Quantification of (+)-catechin, (−)-epicatechin, and procyanidin B1 in PACs-CFE was performed via HPLC analysis (Essentia LC-16, Shimadzu, Kyoto, Japan). Chromatographic separation was carried out on a Sharpsil-AQ C_18_ column (4.6 mm × 250 mm, 5 µm, Xuanmei, Shanghai, China). The mobile phase consisted of 0.1% phosphoric acid in water (A) and acetonitrile (B) at a flow rate of 1.0 mL/min with a detection wavelength of 210 nm. A gradient elution was used as follows: 0–50 min, 5–12% B; 50–90 min, 12–20% B; 90–115 min, 20–95% B; 115–120 min, 95% B.

### 2.4. UPLC-Q-TOF-MS/MS Analysis

The composition of proanthocyanidins in PACs-CFE was analyzed by UPLC-Q-TOF-MS/MS using a quadrupole time-of-flight mass spectrometer (AB SCIEX X500R, AB SCIEX, Singapore). Separation was carried out with an ACQUITY UPLC^®^ HSS T3 column (2.1 mm × 50 mm; 1.8 µm, Waters, Milford, MA, USA) at 0.3 mL/min with UV detection at 210 nm and an injection volume of 5 µL. The mobile phase consisted of 0.1% formic acid in water (A) and acetonitrile (B). The gradient was: 0–20 min, 95–88% A; 20–30 min, 88–80% A; 30–40 min, 80–5% A; 40–50 min, 5% A. MS analysis was performed in negative ion mode.

### 2.5. Cell Culture and Cell Viability Assay

Human hepatic stellate (LX-2) cells were cultured in Dulbecco’s modified Eagle medium (DMEM) supplemented with 10% fetal bovine serum and 1% penicillin–streptomycin at 37 °C in a humidified incubator with 5% CO_2_. For the cell viability assay, LX-2 cells (5 × 10^3^ cells/well) were seeded in 96-well plates and treated with PACs-CFE for 48 h. Cell viability was assessed using the CCK-8 reagent (Goonie, 100–106, Beijing, China) according to the manufacturer’s instructions. The absorbance of each well was measured at a wavelength of 450 nm using a microplate reader (Synergy H1, BioTek, Santa Clara, CA, USA).

### 2.6. Mitochondrial Morphology Measurement

LX-2 cells were seeded in 10 mL culture dishes and divided into the control group and the PACs-CFE treatment (100 μg/mL) group. After 24 h, the culture medium was removed, and cells were fixed with 2.5% glutaraldehyde for 5 min at room temperature in the dark. Cells were then scraped, centrifuged, and resuspended in fresh fixative for an additional 30 min. Samples were stored at 4 °C for 6 h before embedding and sectioning for transmission electron microscopy. For the laser confocal detection, LX-2 cells were seeded in confocal culture dishes and divided into seven groups: blank control and PACs-CFE-treated groups at concentrations of 6.25, 12.5, 25, 50, and 100 μg/mL. After 48 h of treatment, cells were stained with C11-BODIPY (2.5 μM, MCE; Shanghai, China) and Phen Green SK diacetate (2.5 μM; from MCE) along with Hoechst 33342 nuclear stain agent (from MCE). Following 30 min of incubation, cells were washed three times with serum-free medium, followed by capturing cell images using a confocal laser scanning microscope.

### 2.7. Mouse Model of Liver Fibrosis and Sample Collection

Based on preliminary experimental data, to ensure the accuracy of data analysis and adherence to the 3Rs principle, we selected eight mice per group. All animal experiments were approved by the Ethics Committee of Guangdong Pharmaceutical University (Approval No. gdpulacspf2017720). A total of 56 male SPF-grade C57BL/6 mice (6 weeks old, 25–30 g) were purchased from the Guangdong Animal Centre (Guangzhou, Guangdong, China) and housed under standard laboratory conditions (25 °C, 40–60% humidity, 12 h light/dark cycle) with free access to food and water. Mice were randomly grouped into a control group (*n* = 8) and a CCl_4_-treated group (*n* = 48). Mice in the model (CCl_4_-treated) group received intraperitoneal injections of 1 mL/kg CCl_4_ twice weekly for 6 weeks. From week 2, these mice were further divided into subgroups: a model group (saline gavage) and PACs-CFE treatment groups (50, 100, 200, 400, and 800 mg/kg; *n* = 8 for each dosage). Control mice received olive oil intraperitoneally and saline by gavage. All mice were euthanized 48 h after the final dose. Only the feeding personnel know the detailed grouping. Blood and liver tissues from each group were collected for biochemical and histological analyses. The mice were euthanized at the end of the experimental period. Animals were not excluded from this study, and confounders were not controlled. All tissue sections were subjected to quantitative analysis using semi-quantitative image analysis software (ImageJ, version 2.16) to measure parameters such as collagen area and intensity of positive signals.

### 2.8. Zebrafish Liver Fibrosis Model

Wild-type AB strain zebrafish (3 days post-fertilization, dpf) were randomly distributed into 6-well plates (30 fish per well). Liver fibrosis was induced using thioacetamide (TAA), and fish were treated with PACs-CFE at dosages of 15.6, 31.2, and 62.5 μg/mL. Colchicine (7.8 μg/mL) was used as the positive control. All treatments were carried out in 3 mL of solution per well at 28 °C for 72 h. Following treatment, zebrafish were fixed, dehydrated, embedded, and sectioned for reticulin fiber staining and histopathological evaluation.

### 2.9. Serum Biochemistry and Histopathology

Blood samples from mice were collected to evaluate serum biochemical markers including alanine transaminase (ALT), aspartate transaminase (AST), albumin (ALB), alkaline phosphatase (ALP), gamma-glutamyl transferase (GGT), direct bilirubin (DBIL), indirect bilirubin (IBIL), and total bilirubin (TBIL), using commercial assay kits (Macklin, Shanghai, China). Liver tissues were fixed in 4% paraformaldehyde, embedded in paraffin, sectioned, and stained with hematoxylin and eosin (H&E; Solarbio; Beijing, China), Masson’s trichrome, and Sirius Red to assess liver morphology and fibrosis.

### 2.10. Gut Microbiota Analysis

Fecal samples from PACs-CFE-treated and control mice were collected for 16S rRNA gene sequencing. Mice’s microbial DNA was extracted using a commercial DNA extraction kit (Aidlab, Guangzhou, China). DNA purity and concentration were measured with a NanoDrop 2000 spectrophotometer (Thermo Scientific; Waltham, MA, USA), and DNA integrity was verified via 1% agarose gel electrophoresis. The 16S rRNA V3-V4 region was amplified using primers 338F (5′-ACTCCTACGGGGAGGCAGCAG-3′) and 806R (5′-GGACTACHVGGGTWTCTAAT-3′). Polymerase chain reaction (PCR) was performed using the GeneAmp 9700 Thermal Cycler (ABI; Foster City, CA, USA). The PCR had a denaturation for 3 min at 95 °C, followed by cycling for 30 s at 95 °C, annealing for 30 s at 45 °C, and elongation for 45 s at 72 °C. The amplicons were purified and sequenced on the Illumina MiSeq platform (Illumina; San Diego, CA, USA) to construct libraries according to the standard operating procedures.

### 2.11. Reverse Transcription and Quantitative Real-Time PCR (qRT-PCR)

Total RNA was extracted from LX-2 cells using TRIzol reagent (Invitrogen, Guangzhou, China) and reverse-transcribed into cDNA. Gene expression levels were quantified using the 7900HT Real-Time PCR System (Applied Biosystems; Foster City, CA, USA) with gene-specific primers (See Supplementary Table S1). Expression levels were normalized and calculated using the 2^−ΔΔCt^ method.

### 2.12. Western Blotting

Proteins were extracted using RIPA lysis buffer (purchased from MCE) and quantified with a BCA protein assay kit (from Solarbio). Equal amounts of protein were separated on 8% SDS-PAGE gels and transferred to PVDF membranes. After blocking with 5% non-fat milk, membranes were incubated overnight at 4 °C with the primary antibodies, followed by incubation with the secondary antibodies. Protein bands were visualized using an Odyssey imaging system (Li-COR Biosciences; Lincoln, NE, USA), and densitometric analysis was performed using the ImageJ software.

### 2.13. Statistical Analysis

All statistical analyses were performed using GraphPad Prism version 9.0 and Microsoft Excel 2019. Data are presented as mean ± standard deviation (SD). Comparisons between two groups were made using Student’s *t*-test, and one-way analysis of variance (ANOVA) followed by Dunnett’s post hoc test was used for multiple group comparisons. The results of statistical analyses are presented with 95% confidence intervals. A *p*-value < 0.05 was considered statistically significant.

## 3. Results

### 3.1. Proanthocyanidins Composition of PACs-CFE

The total proanthocyanidin content in PACs-CFE was determined to be 84.2%, with an overall extraction yield of 2.5%. We used UPLC-Q-TOF-MS/MS analyses to tentatively identify 38 proanthocyanidin-like compounds in PACs-CFE (Table 1). These compounds are catechins, epicatechins, a series of B-type proanthocyanidins (seven dimers, eleven trimers, and eleven tetramers), three dimeric gallate esters, and four trimeric gallate esters. Compounds with a molecular ion [M–H]^−^ at *m*/*z* 289 were identified as catechins and epicatechins, characterized by MS^2^ fragment ions at *m*/*z* 245 (loss of CO_2_), 205, and 203, consistent with the A-ring cleavage of flavan-3-ols. Ions at *m*/*z* 577 correspond to B-type proanthocyanidin dimers. Their fragmentation yielded *m*/*z* 425 (RDA cleavage), 407 (dehydration of *m*/*z* 425), 451 (A-ring elimination), 559 (loss of water from *m*/*z* 577), and 289 (loss of a catechin/epicatechin unit). Compounds at *m*/*z* 729 were identified as B-type proanthocyanidin dimeric monogallates, with MS^2^ fragments including *m*/*z* 577 (loss of galloyl group), 441 (loss of catechin unit), and 603 (loss of a glucosinol-related moiety). These compounds also produced characteristic fragments at *m*/*z* 425, 407, 451, 559, and 289 [17]. Compounds at *m*/*z* 865 were identified as B-type proanthocyanidin trimers. Fragmentation yielded ions at *m*/*z* 713 (RDA cleavage), 695 (RDA with dehydration), and 577 (interflavan bond cleavage). The trimeric monogallates at *m*/*z* 1017 displayed characteristic fragments at *m*/*z* 891, 847, 729, 695, 603, 559, 451, 407, and 289 [18]. Tetrameric proanthocyanidins were detected at *m*/*z* 1153. MS^2^ analysis revealed fragments at *m*/*z* 1027 (loss of resorcinol unit), 983, 865, 863, and 575, representing successive losses of flavanol subunits [19]. Additionally, we quantified the levels of reported major proanthocyanidins in cili fruit, including (+)-catechin, (−)-epicatechin, and proanthocyanidin B1 [20]. Using a reversed-phase column, we determined the level of (+)-catechin, (−)-epicatechin, and proanthocyanidin B1 in PACs-CFE as 9.9%, 1.9%, and 1.9%, respectively (Table 2).

### 3.2. PACs-CFE Inhibits LX-2 Cell Activation

**Figure 1:**
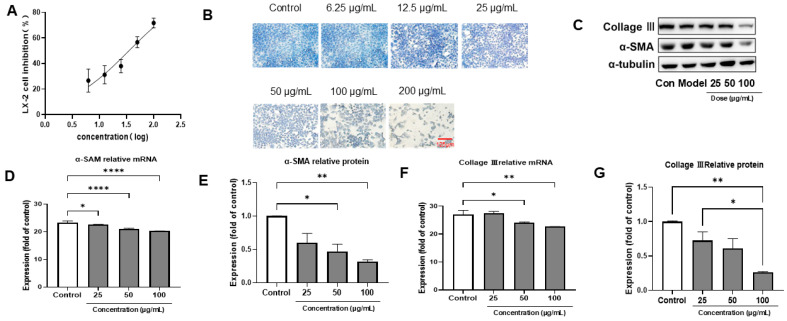
PACs-CFE inhibited the activation of LX-2 cells and attenuated the fibrotic process. (**A**) Inhibition of LX-2 cells treated with different concentrations of PACs-CFE for 48 h; (**B**) Cell growth status of LX-2 cells treated with indicated concentrations of PACs-CFE for 48 h by Masson staining; (**C**) Representative Western blot for collagen III and α-SMA in LX-2 cells treated with indicated concentrations of PACs-CFE for 48 h. (**D**–**G**) Influence of PACs-CFE on fibrogenic gene and protein expressions in LX-2 cells. Assay was carried out in cells treated with indicated concentrations of PACs-CFE for 48 h. The data presented are means  ±  S.D. Statistical significance was determined by Student’s *t*-test. (* *p* < 0.05, ** *p* < 0.01, **** *p* < 0.0001, *n* = 3).

PACs-CFE inhibited the activation of LX-2 hepatic stellate cells in a concentration-dependent manner, with an IC_50_ of 35.2 µg/mL (Figure 1A). Masson’s trichrome staining revealed collagen fibers (blue) in LX-2 cells treated with various concentrations of PACs-CFE. At 50 µg/mL, collagen content was significantly reduced compared to the control. At concentrations above 100 µg/mL, more than 80% of cells exhibited death, and collagen deposition was further reduced (Figure 1B). Collagen III and α-smooth muscle actin (α-SMA), markers of hepatic stellate cell activation, were also suppressed by PACs-CFE. Both protein and mRNA expression levels of collagen III and α-SMA decreased in a concentration-dependent manner (Figure 1C–G), suggesting that PACs-CFE attenuated LX-2 cell activation and may contribute to the inhibition of liver fibrosis progression.

### 3.3. PACs-CFE Alleviates Liver Fibrosis in Zebrafish

**Figure 2:**
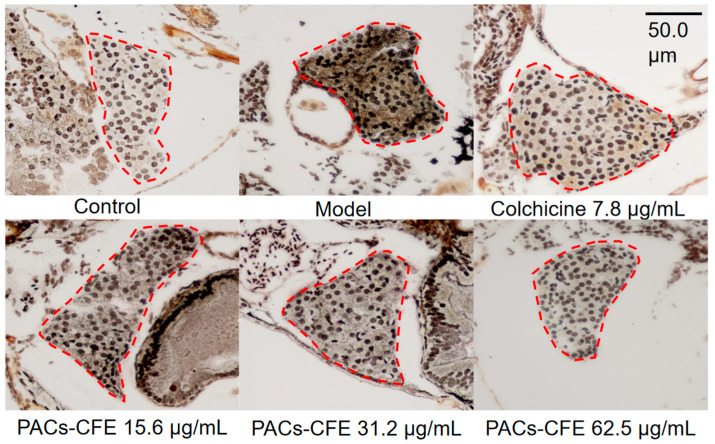
Liver tissue alterations after 72 h treatment of thioacetamide-induced liver fibrosis in zebrafish with indicated concentrations of PACs-CFE and 7.81 µg/mL of colchicine at 28 °C, with zebrafish liver tissue in the red circle. Scale bar, 50.0 μm.

Alterations in the structure of reticular fiber, as the primary scaffolding for hepatocytes and hepatic sinusoids, reflect pathological changes in the liver. Under reticulofiber staining, reticular fibers appeared black, collagen fibers were yellow to yellowish-brown, and cell nuclei were brown to blackish-brown. As shown in Figure 2, the control group displayed normal liver architecture, with clearly defined hepatocyte nuclei arranged in neat rows without observable reticular or collagen fibers. In contrast, the model group exhibited disorganized liver structure, vacuolar degeneration, disordered nuclear arrangement, and a marked increase in reticular and collagen fibers. These characteristics suggest that TAA induced significant fibrotic remodeling in zebrafish. In the positive control group treated with colchicine, liver morphology resembled that of the control group, with clear hepatocyte nuclei and minimal fibrotic fiber deposition. Similarly, treatment with PACs-CFE at 31.2 µg/mL preserved normal liver architecture, showing clearly defined nuclei, absence of vacuolar degeneration, no detectable reticular fibers, and a marked reduction in collagen fibers. These findings suggest that PACs-CFE alleviated liver fibrosis in zebrafish, restoring normal hepatic architecture and reducing fibrotic tissue accumulation.

### 3.4. PACs-CFE Ameliorates Hepatic Fibrosis in Mice

**Figure 3:**
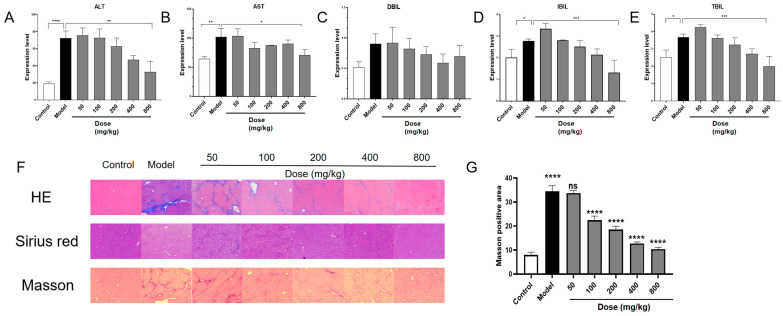
PACs-CFE alleviates the disease process in CCl_4_-induced hepatic fibrosis mice. (**A**–**E**) Effect of indicated concentrations of PACs-CFE on ALT, AST, DBIL, IBIL, and TBIL levels in carbon CCl_4_-induced mice after 6 weeks of treatment; (**F**). Liver tissue alterations after 6 weeks of treatment of CCl_4_-induced liver fibrosis in mice with indicated concentrations of PACs-CFE; (**G**) Effect of indicated concentrations of PACs-CFE on Masson-positive area in CCl_4_-induced mice after 6 weeks of treatment. For (**A**–**E**,**G**), the data presented are means  ±  S.D. Statistical significance was determined by Student’s *t*-test (ns: not statistically significant, * *p* < 0.05, ** *p* < 0.01, *** *p* < 0.001, **** *p* < 0.0001, *n* = 8).

ALT and AST are commonly used serum biomarkers to assess hepatocellular injury, as they are released into the bloodstream when liver cells are damaged. Additionally, bilirubin levels serve as important indicators of liver function, reflecting the liver’s capacity to metabolize and excrete heme breakdown products. Specifically, IBIL must be taken up and conjugated by hepatocytes, while DBIL is the conjugated form that is excreted into bile. When liver function is compromised, the impaired uptake, conjugation, or excretion of bilirubin results in elevated serum levels of IBIL and DBIL. Therefore, changes in ALT, AST, and bilirubin levels provide a comprehensive view of liver injury and the therapeutic potential of PACs-CFE in mitigating fibrosis-related damage. As shown in Figure 3A, the model group (CCl_4_-induced fibrosis) exhibited a significant increase in ALT levels compared to the control group, while the PACs-CFE treatment attenuated this elevation in a dose-dependent manner. Notably, administration of 800 mg/kg reduced ALT levels compared to the model group. Similarly, Figure 3B shows that AST levels followed the same trend as ALT, suggesting that CCl_4_-induced hepatocellular and mitochondrial injury was ameliorated by PACs-CFE, particularly at higher doses. Figure 3C–E further show that TBIL, DBIL, and IBIL were elevated in the model group, whereas PACs-CFE administration reversed these elevations in a dose-dependent manner, with the highest dose restoring bilirubin levels closer to those observed in the control group. Collectively, these results support that PACs-CFE mitigated hepatic injury and fibrosis in a mouse model by improving liver enzyme profiles and restoring bilirubin metabolism.

Histomorphological changes are key indicators for assessing liver fibrosis. The H&E staining assay visualizes liver architecture and inflammatory infiltration, while Sirius red and Masson’s trichrome staining provide evaluation of collagen deposition. As shown in Figure 3F, liver tissue from control mice displayed healthy architecture, with uniform hepatocyte morphology, intact portal areas, and no signs of inflammatory infiltration or fibrotic tissue. In contrast, liver sections from model mice (CCl_4_-induced fibrosis) exhibited disrupted architecture, extensive collagen fiber proliferation forming fibrotic septa, vacuolar degeneration of hepatocytes, and widespread inflammatory infiltration, including lymphocyte aggregation. These characteristics suggest that we have established a mouse liver fibrosis model. Treatment with PACs-CFE improved liver histology in a dose-dependent manner. With increasing doses, hepatocyte arrangement appeared more regular, vacuolar degeneration was reduced, fibrotic septa were diminished, and inflammatory infiltration decreased. Sirius red staining further revealed that collagen fibers in control mice were confined to the perivascular regions, whereas in the model group, collagen extended throughout the lobules, disrupting normal architecture and forming pseudo-lobules. PACs-CFE treatment reduced collagen deposition and preserved lobular structure, with more pronounced effects observed at higher doses. Masson’s trichrome staining further supported these findings. In control mice, minimal collagen depositions were observed around blood vessels. In contrast, the model group showed extensive blue-stained collagen fibers radiating from the vasculature, forming thick fibrotic bands that disrupted lobular organization. PACs-CFE treatment reduced both the intensity and width of these fibrotic bands in a dose-responsive manner. Quantitative analysis of collagen volume fraction in liver tissue confirmed an increase in the model group compared to the control. This condition was reversed by PACs-CFE in a dose-dependent manner (Figure 3G). These findings collectively support that PACs-CFE attenuates hepatic fibrosis in vivo.

Given the observed antifibrotic effects of PACs-CFE in both zebrafish and mouse models, we conducted further studies to elucidate its underlying mechanisms of action. This was achieved by (1) examining whether PACs-CFE can induce ferroptosis in hepatic stellate cells and (2) exploring whether PACs-CFE can modulate gut microbiota composition in the mouse model.

### 3.5. PACs-CFE Induces Ferroptosis in LX-2 Cells

**Figure 4:**
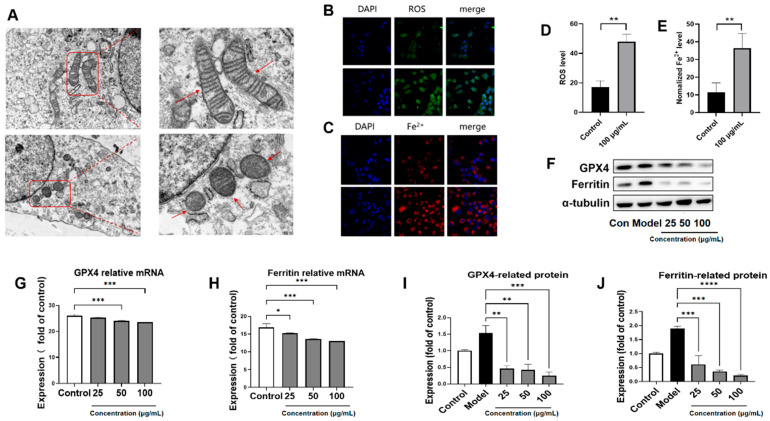
PACs-CFE Induces Ferroptosis in LX-2 Cells (**A**) Electron micrographs of mitochondrial morphology of LX-2 cells treated with 100 μg/mL PACs-CFE for 24 h. Arrows indicate structural abnormalities such as loss of ridge-like structures and mitochondrial atrophy. (**B**–**E**) characterization of ROS and iron levels and fluorescence intensity of LX-2 cells treated with 100 μg/mL PACs-CFE for 24 h; (**F**) Representative Western blot for GPX 4 and ferritin in LX-2 cells treated with indicated concentrations of PACs-CFE for 24 h. (**G**–**J**) Influence of PACs-CFE on GPX 4 and ferritin gene and protein expressions in LX-2 cells. Assay was carried out in cells treated with indicated concentrations of PACs-CFE for 24 h. Data presented are means  ±  S.D. Statistical significance was determined by Student’s *t*-test. (* *p* < 0.05, ** *p* < 0.01, *** *p* < 0.001, **** *p* < 0.0001, *n* = 3).

Ferroptosis, a form of programmed cell death characterized by iron-dependent lipid peroxidation, has been increasingly recognized for its roles in liver fibrosis progression [21,22,23]. Mitochondrial damage is a hallmark of ferroptosis, which can lead to structural abnormalities such as outer membrane rupture, cristae loss, and mitochondrial shrinkage. In this study, we evaluated whether PACs-CFE’s ameliorative effect on liver fibrosis is mediated by inducing ferroptosis in LX-2 Cells. Transmission electron microscopy (Figure 4A) revealed that mitochondria in control LX-2 cells were intact with well-defined cristae. In contrast, PACs-CFE-treated cells exhibited mitochondrial shrinkage, wrinkled membranes, disrupted outer membranes, and reduced or absent cristae. Mitochondria also appeared more electron-dense, while nuclear morphology remained largely unchanged. These observations suggest that PACs-CFE treatment resulted in mitochondrial injury via inducing ferroptosis in hepatic stellate cells.

To further investigate PACs-CFE-induced ferroptosis, we assessed intracellular reactive oxygen species (ROS) and iron ion accumulation using C11-BODIPY 581/589 and Phen Green SK diacetate fluorescent probes. Quantitative image analysis showed significantly increased fluorescence intensities in PACs-CFE-treated cells, indicating elevated ROS levels and iron overload (Figure 4B). These changes suggest enhanced lipid peroxidation and iron accumulation, which is in agreement with the ferroptotic activity. Ferroptosis is driven by dysregulation of the cysteine–glutathione–glutathione peroxidase 4 (GPX4) antioxidant defense system. In this study, PACs-CFE treatment led to a dose-dependent reduction in both protein and mRNA expression of GPX4 and ferritin, which is an iron-storing protein, compared to the control group (Figure 4F–J). These findings suggest that PACs-CFE induced ferroptosis in LX-2 cells by promoting iron accumulation, lipid peroxidation, and suppression of the GPX4-mediated antioxidant pathway. This ferroptotic mechanism may contribute to PACs-CFE’s antifibrotic effects in hepatic stellate cells.

### 3.6. PACs-CFE Modulates the TGF-β1/Smad3 Pathway

**Figure 5:**
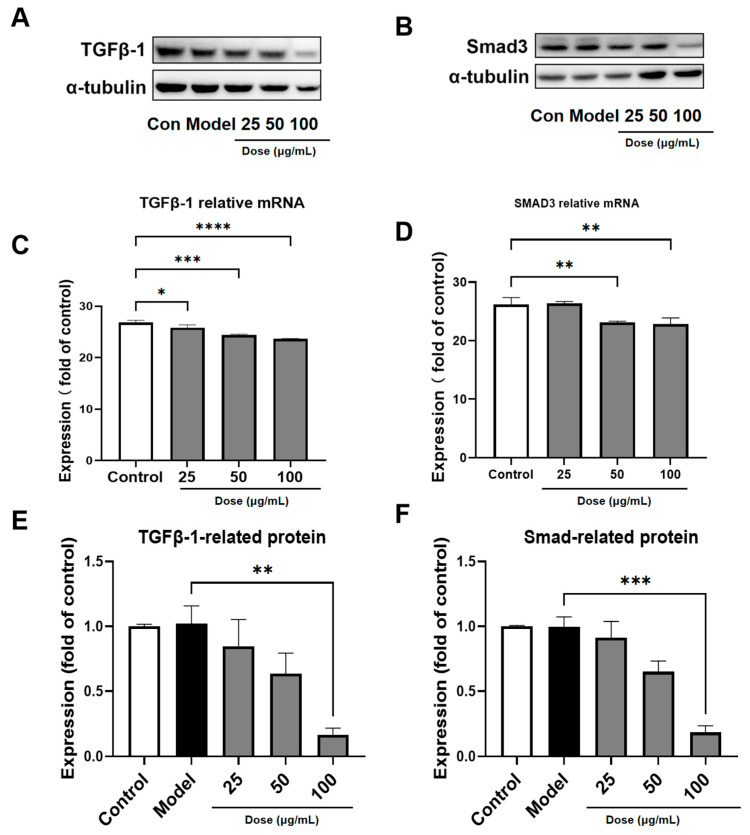
PACs-CFE Modulates the TGF-β1/Smad3 Pathway (**A**,**B**). Representative Western blot for TGF-β1 and Smad3 in LX-2 cells treated with indicated concentrations of PACs-CFE for 48 h. (**C**) Influence of PACs-CFE on TGF-β1 gene expressions in LX-2 cells. (**D**) Influence of PACs-CFE on Smad3 gene expressions in LX-2 cells. (**E**) Influence of PACs-CFE on TGF-β1 protein expressions in LX-2 cells. (**F**) Influence of PACs-CFE on Smad3 protein expressions in LX-2 cells. Assay was carried out in cells treated with indicated concentrations of PACs-CFE for 48 h. The data presented are means  ±  S.D. Statistical significance was determined by Student’s *t*-test. (* *p* < 0.05, ** *p* < 0.01, *** *p* < 0.001, **** *p* < 0.0001, *n* = 3).

The TGF-β1/Smad3 signaling pathway plays a central role in the progression of liver fibrosis. TGF-β1 acts as a key profibrotic cytokine, promoting extracellular matrix deposition and hepatic stellate cell activation, while Smad3 functions as a downstream effector that transduces TGF-β1 signals from the cell membrane to the nucleus via phosphorylation [24,25]. In this study, we found that treatment with PACs-CFE significantly downregulated the expression of both TGF-β1 and Smad3 at the protein and mRNA levels (Figure 5). These findings suggest that PACs-CFE may exert its antifibrotic effects by suppressing the TGF-β1/Smad3 signaling pathway, thereby inhibiting fibrogenic gene expression and hepatic stellate cell activation.

### 3.7. PACs-CFE Modulates Intestinal Microbiota in Mice with Liver Fibrosis

**Figure 6:**
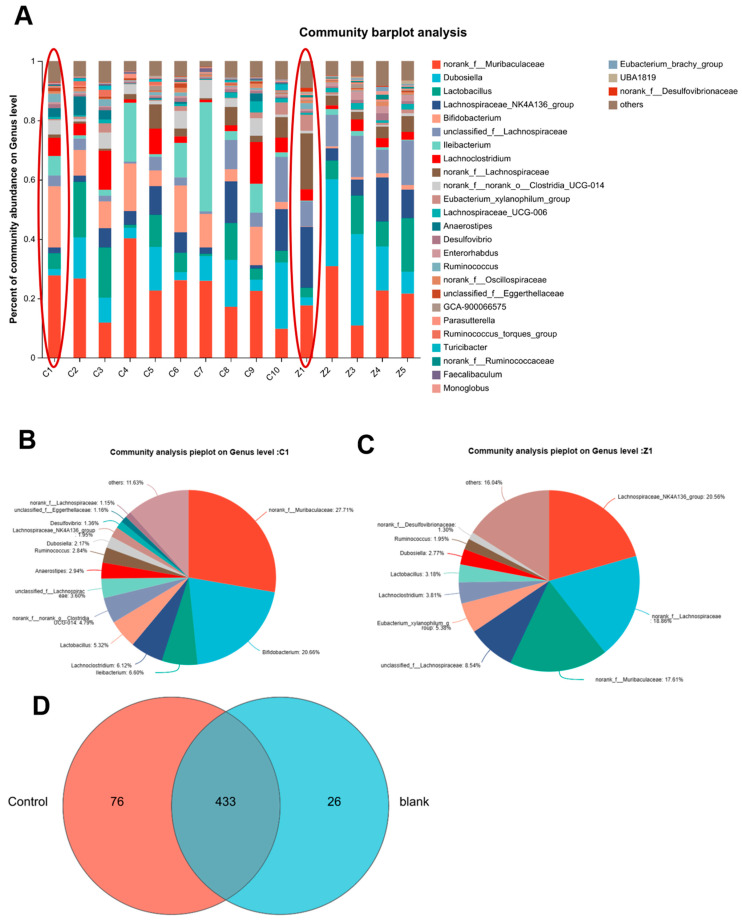
Analysis of intestinal flora in liver fibrosis mice after administration of PACS-CFE. Sample community composition analysis (**A**), species relative abundance histogram ((**B**) is the C1 group within the red circle in (**A**). (**C**) is the Z1 group within the red circle in (**A**)), species relative abundance pie charts (**D**) species Venn analysis plot. For (**A**–**D**), data were collected in CCl_4_-induced hepatic fibrosis mice treated with 400 mg/kg PACs-CFE for 6 weeks.

To assess the effect of PACs-CFE on gut microbiota in liver fibrosis, we analyzed changes in microbial community composition in treated and control mice. A total of 433 operational taxonomic units (OTUs) were shared between the PACs-CFE and control groups, while the administration group exhibited 76 unique bacterial species and a loss of 26 species compared to controls. These changes indicate that PACs-CFE increased overall microbial richness in the intestinal tract (Figure 6). As shown in Figure 6A, the dominant genera in the mouse gut microbiota included *Lactobacillus* and *Dubosiella* among others. Notably, the PACs-CFE-treated group gained beneficial taxa. For instance, several beneficial bacterial genera were enriched in the PACs-CFE-treated group. For instance, *g__norank__f__Muribaculaceae* and *g__Lachnospiraceae_NK4A136_group* are known for their anti-inflammatory effects, ability to promote intestinal mucosal repair, and protective role in colitis models [26,27,28]. *g__Faecalibaculum* contributes to maintaining enzymatic activity and supports the digestive system in defending against intestinal pathogens [29]. Additionally, *g__Prevotellaceae_UCG-001* has been linked to protecting liver function by modulating the AMPK signaling pathway and is negatively correlated with the TC/HDL-C ratio, an indicator of liver health. In contrast, several genera were only found in the control group and are associated with adverse health outcomes. For example, *g__norank__f__norank__o__Clostridia_vadinBB60_group* is positively correlated with serum lipid levels, including high density lipoprotein cholesterol, triglyceride, low density lipoprotein cholesterol, and total cholesterol, as well as fasting blood glucose and body weight gain. *g__Harryflintia* has been negatively correlated with SOD levels in vivo, suggesting a potential link to increased oxidative stress [30]. Furthermore, *g__Erysipelatoclostridium* is notably elevated in patients with Parkinson’s disease and is associated with worsening gastrointestinal dysfunction [31]. These findings suggest that PACs-CFE beneficially remodels the intestinal microbiota in liver fibrosis, enriching for anti-inflammatory and gut-protective taxa while suppressing potentially pathogenic species. This modulation may contribute to its antifibrotic effects by improving gut-liver axis homeostasis.

### 3.8. Functional Prediction of Gut Microbiota and Its Association with Anti-Fibrotic Mechanisms

To explore the potential mechanistic links between intestinal microbiota and hepatic fibrosis, we first addressed the multicollinearity among clinical variables by applying the variance inflation factor (VIF) analysis. Clinical factors with *p* > 0.05 or VIF > 2.0 were excluded to reduce redundancy and improve the robustness of downstream analysis. The filtered variables were then used for distance-based redundancy analysis (db-RDA), and the relationship between microbial taxa and clinical indicators was further visualized using heatmaps to reveal correlation patterns.

**Figure 7:**
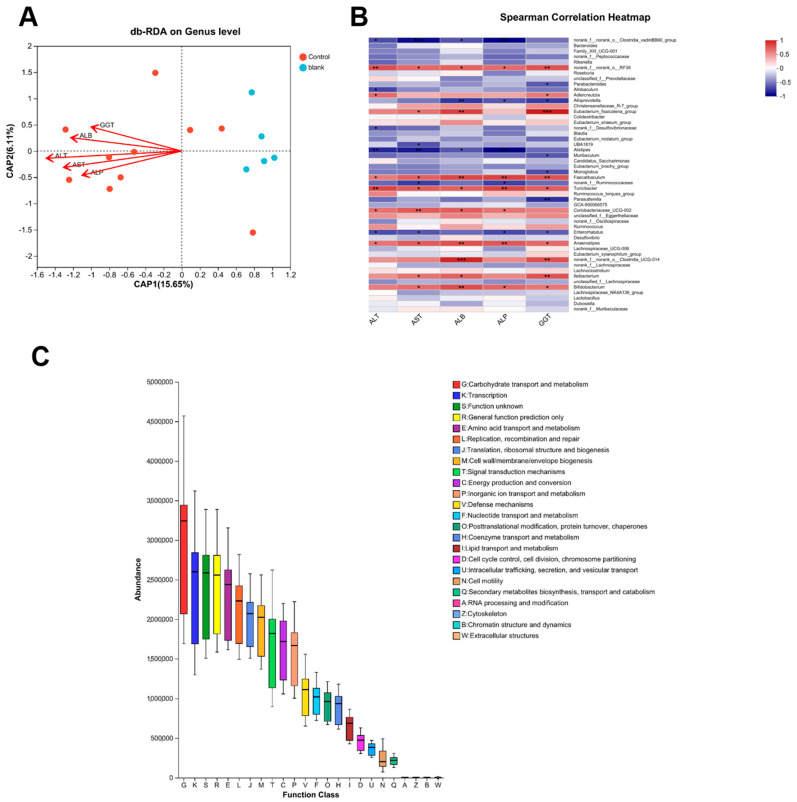
Functional Prediction of Gut Microbiota and Its Association with Anti-Fibrotic Mechanisms. (**A**) flora versus db-RDA clinical factors; (**B**) Heatmap analysis of correlation between flora and clinical factors; (**C**) COG functional enrichment. For (**A**–**C**), data were collected in CCl_4_-induced hepatic fibrosis mice treated with 400 mg/kg PACs-CFE for 6 weeks. (* *p* < 0.05, ** *p* < 0.01, *** *p* < 0.001, *n* = 8).

As shown in Figure 7A, db-RDA and correlation analyses indicated strong associations between gut microbiota composition and key clinical indicators of liver fibrosis, including ALT, AST, and ALB. These findings support that PACs-CFE modulated gut microbial communities and conferred liver beneficial effects. Notably, certain bacterial genera, such as *norank-f-norank-o-RF39*, *Turicibacter*, and *norank-f-norank-o-Clostridia-UCG-014*, were positively correlated with fibrosis-related biomarkers, suggesting a potential pathogenic role. In contrast, genera such as *Alistipes*, *Alloprevotella*, and *norank-f-norank-o-Clostridia-vadinBB60-group* were negatively correlated with fibrosis markers, which suggests that they may have a protective association. Among these, *Clostridia-UCG-014* is recognized as a conditionally pathogenic gut bacterium, while *Alistipes* has been reported to confer protection against hepatic fibrosis, as well as offering benefits in cancer immunotherapy and cardiovascular disease [32]. Thus, PACs-CFE may alleviate liver fibrosis by reshaping the gut microbiota, enhancing the abundance of beneficial microbes, and suppressing opportunistic pathogens. To further explore the functional impact of microbial changes without requiring metagenomic sequencing, we employed PICRUSt to infer metabolic pathway enrichment based on 16S rRNA gene data. The OTU abundance table was normalized and annotated with clusters of orthologous groups (COG) and Kyoto encyclopedia of genes and genomes (KEGG) functions using Greengenes IDs. As shown in Figure 7C, the COG function prediction revealed that gut microbiota may influence disease progression through pathways related to carbohydrate transport and metabolism, transcription, amino acid transport and metabolism, replication, recombination and repair, and translation/ribosomal structure and biosynthesis. These functions are closely associated with cellular homeostasis and immune regulation, further supporting the role of PACs-CFE in modulating host–microbiota interactions as part of its anti-fibrotic mechanism.

## 4. Discussion

In this study, we demonstrated that PACs-CFE, a proanthocyanidin-rich extract derived from cili fruit using macroporous resin column chromatography, exerted significant anti-fibrotic activity. This was supported by its inhibitory effects on LX-2 hepatic stellate cell activation and its ameliorative effects against hepatic fibrosis in both a TAA-induced zebrafish model and a CCl_4_-induced mouse model. Moreover, PACs-CFE may exert its effects through multiple pathways, including ferroptosis induction in hepatic stellate cells, suppression of the TGF-β1/Smad3 signaling cascade, and modulation of gut microbiota composition.

Chemical analysis revealed that PACs-CFE contained 84.2% total proanthocyanidins, comprising 38 structurally diverse components identified for the first time from cili fruit using UPLC-Q-TOF-MS/MS. These included catechins, epicatechins, and a range of B-type proanthocyanidin dimers, trimers, tetramers, and gallate esters. Although several studies have reported the potential health benefits of cili fruit extracts, including anti-inflammatory and liver protective activities [33,34], the specific phytochemical constituents responsible for these effects have remained largely undefined. In addition to structurally characterizing the proanthocyanidin profile of cili fruit, this study is the first to evaluate its anti-fibrotic effects in both cellular and animal models. Proanthocyanidins are known for their hepatoprotective properties, including suppressing hepatic stellate cell activation via fibrogenic pathways such as Hedgehog signaling [35,36]. The superior inhibitory activity of PACs-CFE to crude cili fruit juice suggests that these proanthocyanidins are a critical component for mediating the anti-fibrotic effects. By establishing the link between defined phytochemicals and their biological activity, this study provides valuable insights into the active components of cili fruit. Moreover, the identification and validation of PACs-CFE as an effective anti-fibrotic extract supports its potential as a standardized, compound-specific ingredient for the development of cili dietary supplements targeting liver health.

Hepatic fibrosis is a progressive condition with limited treatment options and substantial side effects associated with current therapies [37]. During fibrosis, hepatic stellate cells become activated and secrete excess extracellular matrix, leading to scar formation. Our findings showed that PACs-CFE reduced collagen and α-SMA expression in a concentration-dependent manner in LX-2 cells, suggesting its ability to suppress fibrogenic activation. In the CCl_4_-induced mouse model, PACs-CFE mitigated liver damage, restored normal hepatic architecture, reduced inflammatory cell infiltration and lymphocyte aggregation, and improved serum biochemical markers, including ALT, AST, and bilirubin. These effects were corroborated in the zebrafish model, suggesting consistent anti-fibrotic efficacy across species. However, several limitations should be acknowledged. Although the LX-2 cell line is a widely accepted model for studying hepatic stellate cell activation, it represents a simplified in vitro system that does not fully recapitulate the complexity of liver fibrosis in vivo. Similarly, while the zebrafish and CCl_4_-induced mouse models provided valuable information, they do not capture the full heterogeneity of human liver fibrosis, which can arise from diverse etiologies. Moreover, the long-term safety, pharmacokinetics, and optimal dosing of PACs-CFE have not yet been assessed. To address these limitations, future research should evaluate PACs-CFE in additional disease-relevant models. Comprehensive pharmacokinetic profiling, toxicity assessments, and mechanistic studies using human-derived cells or liver organoids are critical to support clinical translation.

Notably, PACs-CFE induced morphological and biochemical hallmarks of ferroptosis in LX-2 cells, including mitochondrial shrinkage, loss of cristae, and increased intracellular levels of ROS and iron. These changes were accompanied by downregulation of GPX4 and ferritin, key regulators of ferroptotic resistance [38,39,40]. This suggests that PACs-CFE induces ferroptosis in hepatic stellate cells, contributing to its anti-fibrotic effects through selective elimination of activated HSCs. We also observed that PACs-CFE downregulated both protein and mRNA expression of TGF-β1 and Smad3 in a concentration-dependent manner. Given the central role of the TGF-β1/Smad3 pathway in promoting hepatic fibrosis through ECM accumulation, these findings support PACs-CFE’s role in attenuating fibrogenic signaling [41,42].

Beyond cellular mechanisms, PACs-CFE also influenced the gut–liver axis, a critical modulator of hepatic health [43]. Disruption of gut microbiota has been implicated in liver fibrosis and cirrhosis due to bacterial translocation and systemic inflammation. In our study, CCl_4_-induced liver fibrosis in mice was associated with decreased microbial diversity and a shift toward pathogenic taxa. PACs-CFE treatment restored microbial richness and composition, increasing the abundance of beneficial genera such as *Muribaculaceae*, *Lachnospiraceae_NK4A136*, *Faecalibaculum*, and *Prevotellaceae_UCG-001*, while reducing opportunistic pathogens like *Clostridia_vadinBB60* and *Erysipelatoclostridium*. These microbial changes were in agreement with improvements in liver injury markers (i.e., ALT, AST, ALB), suggesting that PACs-CFE’s liver protective effects were mediated by the host-microbiota interactions. However, the study’s reliance on 16S rRNA sequencing limits functional resolution of microbial pathways, and causality between specific microbial shifts and anti-fibrotic outcomes remains to be confirmed. Future work should integrate metagenomic or metabolomic profiling and microbiota transplantation experiments to better elucidate the functional role of gut microbes in mediating the effects of PACs-CFE.

Based on the above results, PACs-CFE exhibits anti-hepatic fibrosis effects, mitigating carbon tetrachloride-induced liver injury. This effect is similar to that of various flavonoid components, such as grape see [4], *Scorzonera austriaca* Wild [44]. PACs-CFE exhibits a broader safety margin, demonstrating anti-fibrotic effects at a dose as low as 50 mg/kg. Therefore, we propose that PACs-CFE is likely to serve as a potential material for the drugs against chemical hepatic injury.

## 5. Conclusions

To date, this is the first study using in vitro and in vivo models to evaluate the anti-fibrotic activity of a proanthocyanidin-rich extract from cili fruit. By integrating phytochemical profiling, cell-based assays, in vivo models, and microbiota analyses, our findings support that PACs-CFE may exert protective effects against liver fibrosis via the mediation of hepatic stellate cell inactivation, ferroptosis induction, suppression of the TGF-β1/Smad3 signaling pathway, and modulation of gut microbiota composition. These results highlight PACs-CFE as a multi-targeted natural product for liver health and provide mechanistic insight into its bioactivity at the molecular, cellular, and microbiome levels. Furthermore, this study provides characterization of bioactive proanthocyanidins from cili fruit, contributing valuable information for its development as compound-specific functional ingredients. Future research is warranted to validate these findings in clinically relevant models, which is critical for exploring the potential of PACs-CFE as a novel dietary supplement for the management of hepatic fibrosis.

## Figures and Tables

**Table 1:** Tentatively identified phytochemicals of PACs-CFE by UPLC-Q-TOF-MS/MS.

NO	RT/min	*m*/*z* [M-H]-	Identification	Molecular Formula	MS^2^ Ions (*m*/*z*)	ppm
1	1.88	865.1974	B-type proanthocyanidin trimer	C_45_H_38_O_18_	865.1976, 739.1680, 695.1403, 577.1363, 407.0780, 287.0564	−1.3
2	4.12	865.1978	B-type proanthocyanidin trimer	C_45_H_38_O_18_	865.1985, 739.1697, 695.1416, 577.1350, 407.0778, 287.0564	−0.9
3	5.44	577.1344	B-type proanthocyanidin dimer	C_30_H_26_O_12_	577.1346, 451.1033, 425.0876, 407.0766, 289.0712, 255.0301, 125.0242	1.3
4	6.39	865.1975	B-type proanthocyanidin trimer	C_45_H_38_O_18_	865.2011, 739.1680, 695.1409, 577.1373, 407.0776, 287.0564	−1.2
5	7.05	865.1989	B-type proanthocyanidin trimer	C_45_H_38_O_18_	865.1953, 739.1672, 695.1398, 577.1337, 407.0770, 287.0556	0.4
6	7.07	1153.2619	B-type procyanidin tetramers	C_60_H_50_O_28_	1153.2646, 1001.2092, 983.2072, 865.1980, 575.1198, 449.0911, 739.1715	0
7	7.8	577.1352	B-type proanthocyanidin dimer	C_30_H_26_O_12_	577.1343, 451.1019, 425.0911, 407.0779, 289.0709, 287.0564, 245.0825, 125.0240	0.2
8	8.16	865.1974	B-type proanthocyanidin trimer	C_45_H_38_O_18_	865.1953, 739.1672, 695.1398, 577.1337, 407.0770, 287.0556	−1.3
9	8.52	1153.2621	B-type procyanidin tetramers	C_60_H_50_O_28_	1153.2640, 1001.2169, 983.2226, 865.1972, 575.1209, 449.0897, 739.1673	0.2
10	9.49	865.1982	B-type proanthocyanidin trimer	C_45_H_38_O_18_	865.1977, 739.1671, 695.1415, 7.1354, 407.0781, 287.0564	−0.4
11	9.6	1153.2608	B-type procyanidin tetramers	C_60_H_50_O_28_	1153.2585, 1001.2150, 983.2022, 865.1967, 575.1186, 449.0878, 739.1668	−1
12	9.68	289.0719	catechins	C_15_H_14_O_6_	245.0796, 205.0503, 203.0722	0.5
13	10.2	865.1979	B-type proanthocyanidin trimer	C_45_H_38_O_18_	865.1957, 739.1660, 695.1402, 577.1344, 407.0767, 287.0559	−0.7
14	10.67	1153.2627	B-type procyanidin tetramers	C_60_H_50_O_28_	1153.2591, 1001.2169, 983.2085, 865.1984, 575.1204, 449.0885, 739.1670	0.7
15	11.17	577.1341	B-type proanthocyanidin dimer	C_30_H_26_O_12_	577.1354, 451.1041, 425.0876, 407.0767, 289.0713, 299.0567, 125.0241	1.7
16	11.44	1153.2625	B-type procyanidin tetramers	C_60_H_50_O_28_	1153.2602, 1001.2149, 983.2193, 865.1972, 739.1685, 575.1201, 449.0879	0.5
17	11.7	865.1987	B-type proanthocyanidin trimer	C_45_H_38_O_18_	865, 1971, 739.1665, 695.1405, 577.1361, 407.0775, 287.0560	0.2
18	11.81	729.1456	B-type proanthocyanidin dimeric monogallate	C_52_H_42_O_22_	729.1448, 577.1343, 425.0876, 407.0770, 287.0568	0.7
19	12.13	1153.2622	B-type procyanidin tetramers	C_60_H_50_O_28_	1153.2621, 1001.2182, 983.2076, 865.2001, 739.1703, 575.1201, 449.0877	0.2
20	12.42	1017.2082	B-type proanthocyanidin trimeric monogallate	C_52_H_42_O_22_	1017.2063, 891.1785, 865.1923, 847.1709, 729.1449, 577.1339, 407.0770, 287.0554	−1.3
21	12.47	577.135	B-type proanthocyanidin dimer	C_30_H_26_O_12_	577.1334, 425.0869, 407.0759, 289.0712, 287.0555, 125.0244	0.3
22	13.2	1153.2618	B-type procyanidin tetramers	C_60_H_50_O_28_	1153.2601, 1001.2159, 983.2059, 865.1971, 739.1668, 575.1210, 449.0888	−0.1
23	14.24	1153.2611	B-type procyanidin tetramers	C_60_H_50_O_28_	1153.2592, 1001.2146, 983.2041, 865.1991, 739.1683, 575.1199, 449.0882	−0.7
24	15.27	1153.2611	B-type procyanidin tetramers	C_60_H_50_O_28_	1153.2595, 1001.2140, 983.2060, 865.2001, 739.1672, 575.1186, 449.0874	−0.7
25	15.73	729.1443	B-type proanthocyanidin dimeric monogallate	C_37_H_30_O_16_	729.1442, 577.1326, 425.0862, 407.0756, 287.0707	2.5
26	16.55	577.1339	B-type proanthocyanidin dimer	C_30_H_26_O_12_	577.1579, 407.0772, 301.0709, 289.0711, 245.0822, 125.0245	2.2
27	17.78	729.1465	B-type proanthocyanidin dimeric monogallate	C_37_H_30_O_16_	729.1479, 577.1335, 425.0882, 407.0771, 287.0566, 289.0719	0.5
28	17.88	865.1976	B-type proanthocyanidin trimer	C_45_H_38_O_18_	865.1969, 739.1683, 695.1398, 577.1354, 407.0770, 287.0559	−1.1
29	18.67	1153.2612	B-type procyanidin tetramers	C_60_H_50_O_28_	1153.2612, 1001.2169, 983.2032, 865.1998, 739.1681, 575.1200, 449.0866	−0.6
30	18.97	1017.2103	B-type proanthocyanidin trimeric monogallate	C_52_H_42_O_22_	1017.2092, 891.1809, 865.1638, 847.1553, 729.1460, 729.1460, 577.1375, 407.0823, 287.0563	0.8
31	19.21	865.197	B-type proanthocyanidin trimer	C_45_H_38_O_18_	865.1950, 739.1665, 695.1412, 577.1360, 407.0764, 287.0560	−1.8
32	19.65	729.146	B-type proanthocyanidin dimeric monogallate	C_37_H_30_O_16_	729.1470, 577.1356, 425.0866, 407.0768, 289.0716, 287.0548	0.2
33	19.77	1153.2617	B-type procyanidin tetramers	C_60_H_50_O_28_	1153.2602, 1001.2171, 983.2070, 865.2009, 739.168, 575.1202, 449.0862	0.2
34	20.32	865.1972	B-type proanthocyanidin trimer	C_45_H_38_O_18_	865.1972, 739.1679, 695.1400, 577.1354, 407.0770, 287.0564	−1.5
35	21.01	577.1344	B-type proanthocyanidin dimer	C_30_H_26_O_12_	577.1761, 407.0777, 289.0714, 287.0567, 245.0474, 125.0236	1.3
36	21.5	289.0719	epicatechin	C_15_H_14_O_6_	245.0817, 205.0483, 203.0698	0.5
37	24.43	1017.2098	B-type proanthocyanidin trimeric monogallate	C_52_H_42_O_22_	1017.2080, 891.1795, 865.1624, 847.1614, 729.1460, 577.1349, 407.0769, 287.0556	0.3
38	30.88	577.135	B-type proanthocyanidin dimer	C_30_H_26_O_12_	577.1365, 425.0942, 289.0715, 287.0562, 125.0241, 245.0816, 407.0773	0.2

**Table 2:** Phytochemical profile of PACs-CFE.

	PACs-CFE
The total proanthocyanidin (%)	84.2
Proanthocyanidin B1 (%)	1.9
(+)-catechin (%)	9.9
(−)-epicatechin (%)	1.9

## Data Availability

The data that support the findings of this study are not openly available due to reasons of sensitivity and are available from the corresponding author upon reasonable request.

## Supplementary Materials

The following supporting information can be downloaded at: https://www.mdpi.com/article/10.3390/nu17213463/s1, Table S1: Gene-specific primers for the 7900HT real-time PCR system.
