# Supplementary material for: A Proanthocyanidins-Rich Cili (Rosa roxburghii) Fruit Extract Protects CCl4-Induced Mouse Hepatic Fibrosis via Modulation of Ferroptosis and Gut Microbiota
Source: Nutrients. 2025 Nov 3;17(21):3463. doi: 10.3390/nu17213463 (PMC12609504; doi:10.3390/nu17213463)
Supplement: Supplementary file 1 [file nutrients-17-03463-s001.zip › nutrients-3914837-supplementary.pdf]

Supplement Table S1: Gene-specific primers for the 7900HT real-time PCR system

Gene	Sequences（5′-3′）	Lengths（bp）
Collagen III	F: TGAAGGGCAGGGAACAACTTGATG	143
	R: GGATGAAGCAGAGCGAGAAGTAGC	
α-SMA	F: CTTCGTTACTACTGCTGAGCGTGAG	139
	R: CCCATCAGGCAACTCGTAACTCTTC	
GPX4	F: CCGCTGTGGAAGTGGATGAAGATC	115
	R: CTTGTCGATGAGGAACTGTGGAGAG	
Ferritin	F: TTCAACAGTGCTTGGACGGAACC	90
	R: AGGGTGCGGTGAAGAGGTGAC	
TGFβ-1	F: TATTGAGCACCTTGGGCACTGTTG	135
	R: CCTTAACCTCTCTGGGCTTGTTTCC	
Smad3	F: AGGACACAGGAAGAGACGGAAGG	150
	R: CACACCAGGCACATACTTCAGACTC	
